# Quantifying Contact with the Environment: Behaviors of Young Children in Accra, Ghana

**DOI:** 10.4269/ajtmh.15-0417

**Published:** 2016-04-06

**Authors:** Peter F. M. Teunis, Heather E. Reese, Clair Null, Habib Yakubu, Christine L. Moe

**Affiliations:** Center for Global Safe Water, Sanitation, and Hygiene, Hubert Department of Global Health, Rollins School of Public Health, Emory University, Atlanta, Georgia; Centre for Zoonoses and Environmental Microbiology, Centre for Infectious Disease Control, National Institute for Public Health and the Environment, Bilthoven, The Netherlands

## Abstract

To better understand the risks of exposure for young children to fecal contamination in their environment, we systematically characterized and quantified behaviors of 154 children, 0–5 years old, in four high-density, low-income neighborhoods in Accra, Ghana. A repertoire of six different activities and five different compartments (categories of locations within the household) was developed, and about 500 hours of ordered structured observations of activities and locations of individual children were collected. These records were analyzed using a competing hazards model, estimating (Weibull) hazard rates for each state (activity/compartment combination), dependent on the present state and the preceding state. The estimated rates were used to simulate sequences of behavior and describe days in the life of a child in low-income, urban Africa. Children younger than 1 year spent most time playing or sleeping off the ground, older children frequently played on floors. Relatively little time was spent in drains or wet trash areas. Critical combinations of activities, like handwashing after defecation or before eating were estimated to occur rarely. These quantitative behavior estimates can inform future risk assessments that examine the relative roles of various fecal–oral exposure pathways in low-income urban settings.

## Introduction

Diarrheal illness is an important cause of childhood morbidity and mortality worldwide,^1^ especially in countries where many people do not have access to safe water and sanitation.^2^ In western Europe and the United States, decreasing child mortality and increasing life expectancy have been attributed to dramatic improvements in sanitation,^3^ in response to the threat of cholera and typhoid in densely populated nineteenth-century cities.^4^ Improved hygiene decreases infection pressure for environmentally transmitted diseases.^5^ Recently, child contact with a fecal contaminated environment has also been linked to environmental enteropathy.^6^

Recent studies have examined the impact of water, sanitation, and hygiene interventions in low-income countries and documented decreased incidence of diarrheal illness after interventions in sanitation and/or hygiene behavior.^7^,^8^ Interventions in sanitation and/or hygiene usually lead to a reduction of 30–50% in incidence of diarrhea.^9^ Importantly, studies that combined multiple interventions did not find correspondingly stronger effects. This absence of additivity in combinations of more than one intervention has been explained as the consequence of different, competing pathways of exposure.^10^

In an environment where fecal contamination is common, exposure to fecal pathogens is likely to result from more than a single source^11^: food and water may be contaminated, and both indoor and outdoor surfaces may harbor fecal matter. Furthermore, children playing outdoors may come into contact with contaminated environments like open wastewater drains, open defecation sites, or contaminated surface waters. Contact with any one of these environments may cause substantial risk of infection, so that contact with another contaminated environment may not increase the risk much more. In such high-risk environments, the contributions of multiple pathways to the risk may be quantified through exposure: the numbers of pathogens (or the amount of fecal matter) that a child may ingest within a specified period, such as 1 day. To quantify exposure, one needs to know the concentrations of pathogens (or feces) in the environmental compartments that a child has contact with and the intensity of contact with that contaminated media.

The study reported here is a part of the SaniPath study^12^ that aims to characterize the risk of exposure to fecal contamination from different transmission routes, using a risk-based (bottom up) approach: quantify both the presence of fecal pathogens in the environment and the contacts of young children with the fecal-contaminated environment. This article deals with the second problem: behaviors associated with potential contact with fecal contamination, and in particular addresses the question—can these behaviors be systematically quantified? First, it is necessary to define a set of behaviors that is relevant for contact with fecal matter. Then, the selected behaviors must be quantified: for some exposure pathways, the frequency of an activity is relevant (hands touching a contaminated surface, for instance); or other pathways, the duration of the activity is more relevant (playing on a contaminated floor, for instance).

We started with a description of the collected behavioral observations, and showed how to visualize the collected data and identify patterns relevant for hygiene. We then proceeded to develop a mathematical model that systematically describes and quantifies behaviors for use in quantitative assessment of exposure to fecal pathogens. Potential uses for the model are discussed by describing various outputs relevant for predicting child behavior and enteric disease risk. Outcomes like frequency, duration, and sequences of activities are compared for children of different ages living in different neighborhoods.

### Behavioral observations.

Structured observation data on hygiene-related behavior of young children were collected by trained observers, aiming to record all activities of a single child during a period of about 6 hours starting from 6 am to 12 pm or from 12 pm to 6 pm. Structured observation data were collected from March to August 2012. Data were entered using standardized forms, designed by an expert team and tested in a pilot study. To guarantee consistency in data collection across the four observers, they had received prior training, and group meetings with the supervisor were held at the end of each day to discuss how to record specific behaviors. Completed forms were double entered into a digital database, and any discrepancies between entries were checked by a third individual against the paper forms. Observations were ordered into four nested levels: 1) neighborhoods, 2) environments within neighborhoods (household, nursery, school, beach, …), 3) compartments within environments (on floor, off ground with caregiver, …), and 4) behaviors occurring within compartments. Within a neighborhood, a child can be in any one of a set of environments. In this article, the observations from households and nurseries are studied as the main environments for children 0–5 years of age.

The study households were located in four low-income, high-density neighborhoods in urban Accra. Households in these neighborhoods generally comprise a single room (approximately 16 m^2^) that collectively form compound houses made from permanent and semipermanent materials. In the poorest study households, a single room may open directly on to an alley. The study child would generally sleep in the room, but all other activities—bathing, eating, and playing, occurred in the public domain of the alley. The wealthier study households often lived in compounds that consisted of a series of rooms around a central courtyard. Extended families with multiple households would share the courtyard space. The rooms were primarily used for sleeping, whereas eating, bathing, and playing occurred in the courtyard.

Within any environment, there is a set of different compartments (categories of locations) in which exposure behaviors may occur. For this study, setting the list of five compartments is exhaustive: any child must always reside in one of these compartments (Table 1).

Key characteristics of the domestic environment included floors that were usually concrete (improved floor), but dirt floors (unimproved floors) were often observed in the poorest households. The courtyards or alleys were concrete, dirt, or a mix of both floors. When young children were off the ground, they were either on a bed or crib, sitting on a chair, or sitting on the lap of an adult or older child. Most household compounds included areas where there was stagnant water on the ground—either around water taps or laundry/bathing areas. Household trash was sometimes scattered around the courtyard or accumulated in a corner of the courtyard. We designated areas of stagnant water and/or accumulated trash as one type of exposure compartment that may contain human and animal feces due to behaviors we observed associated with fecal exposure. These stagnant water/trash areas also occurred frequently in the public domain of the neighborhood. Open drains were ubiquitous in the study neighborhoods and lined the side of every street or were on the side or center of alleys. These drains contained household wastewater (both graywater and blackwater from flush or pour-flush toilets), storm runoff from the street, trash, dumped excreta from child feces stored in potties and excreta from open defecation.

We assume that any child may exhibit a defined repertoire of six activities when it is in any of the studied environments (different environments may be associated with different behaviors). These activities were chosen by a team of sanitation and health experts. Each of the observed activities may occur in different compartments within an environment. Note that, for very young children, activities like handwashing, bathing, and eating involve a caregiver (parent, older sibling, or other member of the household).

A set of behavioral observations consists of individual records, each with observations of any activity in any compartment, as a sequence observed within an observation period (the target period was about 6 hours, starting at either 6 am or 12 pm). Observations started with noting the time, the current activity, and the current compartment where that activity was observed. Any change in behavior, either a new activity or a change of compartment or both, initiated the next observation: the time of change was recorded as well as the new activity and compartment. This was continued until the end of the observation period. An illustration of the resulting data is shown in Table 2.

Four low-income neighborhoods in Accra, Ghana (Alajo, Bukom, Old Fadama, and Shiabu) were selected for the SaniPath study to capture a diverse set of conditions including squatter and formal settlements (where residents have tenure over their land), coastal and inland areas, frequency of flooding, sanitation coverage, age of neighborhood, proximity to schools and markets, and one mixed income neighborhood. The characteristics of these neighborhoods are further described elsewhere.^13^ Data were collected from households and nurseries.

Households were selected by neighborhood liaisons based on selection criteria. The criteria required that households should have at least one child under 5 years and capture varying levels of child mobility and sanitation facilities. At the time of observation, the child was required to be in good health and engage in normal activity. To achieve a balanced spatial distribution, each neighborhood was split into four sections using known local boundaries. Ten eligible households were selected every Friday from one section for observation on the following week. Informed consent was obtained from each household before the day of observation. Each eligible child was assigned a number and on the day of observation a random drawing of assigned numbers was conducted to select the child to be observed. Numbers of children observed and ranges of numbers of observations per child are given in Table 3. As the majority of observations were collected in households, this article will focus mainly on behaviors within households. Selection of households was nonrandom, but based on achieving a broad range of child mobility, sanitary conditions, and locations within the neighborhoods. Nevertheless, in the following analysis, we have assumed that these household samples are representative of the population in these neighborhoods.

The study was reviewed and approved by the Institutional Review Board at Noguchi Memorial Institute for Medical Research (University of Ghana) and Emory University.

### Analysis of behavioral data.

For brevity, let us call the current activity/compartment combination the *state* of the subject, and a change of state an *event*. Although the data set contains many observations (in total 1,684 events in the households and 162 in the nurseries), many states were rarely observed. The record of sequences of states, corresponding to transitions in behavior and/or compartment thus is also sparse.

Observed states, and transitions between those states, may be visualized as a directed, weighted network, where nodes represent states and edges represent transitions. The frequency with which a transition is observed may be used as the weight of the corresponding edge. Such networks can be conveniently visualized and analyzed using the R–package igraph.^14^

In addition to the state of a subject, the duration between events was also recorded and contains relevant information. Therefore, instead of counting occurrences of observed states, we estimated rates of changes between states, that is, transitioning from any state to any other state.

### Estimating rates of behavior.

The duration of each observed state is assumed to be a realization of a random process in continuous time. From the onset of any present state, all possible subsequent states compete for the next state, each with their own hazard function.^15^,^16^ Thus, for any duration of the present state, the likelihood of any subsequent state may be calculated, dependent on the hazard rate (which is a scale parameter for the Weibull hazard we have used). Obviously, each transition, defined by present and subsequent states, needs its own rate estimate. The rate of moving from “sleeping off ground” to “playing on a dirt floor” is assumed to be different than the rate of moving from “sleeping off ground” to “eating off ground.” In fact, when assessing the marginal durations of states (that is the total time spent in states) it became obvious that it is necessary to also take into account what the state immediately preceding the present state was. The rate of moving from “sleeping off ground” to “playing on a dirt floor” to “eating off ground” is different from “eating on a dirt floor” to “playing on a dirt floor” to “eating off ground”. Inclusion of ancestors and descendants of states was sufficient to provide good estimates of marginal durations of states. It may be noted that addition of higher order dependencies, that is longer chains of states, are theoretically possible, but lead to very complicated calculations.

Because any state is defined by the combination of an activity and the compartment where this activity occurred, there are 5 × 6 = 30 different states (Table 1). As each rate is determined by both the present and the previous states, it follows that for each of the four neighborhoods, a set of 30 × 30 scale parameters must be estimated.

The information about all possible states (i.e., compartment/activity combinations) is incomplete. Some states were not observed because they are not meaningful (like sleeping in a drain) or so rare that they were never observed. States that were not included were: sleeping on a dirt floor, sleep, wash hands, bathe, or defecate in a drain or stagnant water/trash area, or eating in a drain. Some states are rare and may be observed only once in one of the four neighborhoods. Such rare, but not impossible, states were included in the model, producing estimates (low rates) also for the neighborhoods in which such states were not observed.

Details of the mathematical model may be found in the Supplemental Appendix.

We need to be able to generalize from the observed patterns of behavior, by generating a Monte Carlo sample of a sequence of activities and compartments, and thus describe a typical “day in the life of a child” in a low-income, African urban neighborhood.

Using the estimated transition rates, sequences of states can be simulated by random sampling of durations of states, conditional on their ancestor states, and selecting the state with the shortest duration as the next descendant state. This can be repeated as often as desired to calculate, for example, summary statistics for simulated child behaviors or network graphs showing patterns in behavior sequences. All simulations started with sleeping off ground, and then proceeded for a total duration of 14 hours (assumed waking period for a child). The statistics shown below are all based on a simulated population of 1,000 children.

## Results

**Figure 1.**
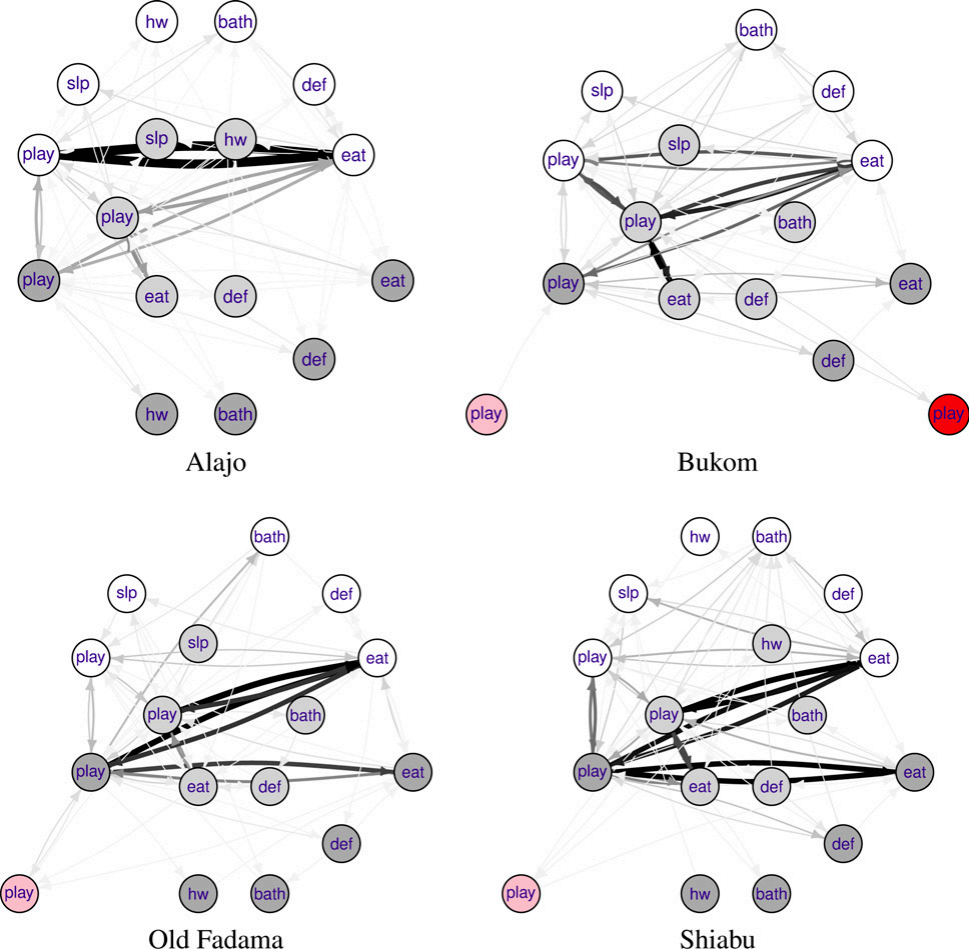
Observed states of children less than 5 years of age in households in the four neighborhoods, and observed transitions among these states. Behaviors: play, slp (sleep), hw (washing hands), bath (bathing), def (defecating), eat. Compartments off ground (white), concrete floor (light gray), dirt floor (dark gray), wet garbage area (pink), drain (red). Arrows indicate transitions between states, strengths (numbers of times the transition was observed) indicated by arrow width and shade (darker lines indicate higher frequency).

Records of structured observations contain information about how children move between states (activity–compartment pairs). It is useful to visualize such data as directed networks with weighted edges (Figure 1

). There are a few states (play and eat) that occur most frequently (Figure 2

**Figure 2.**
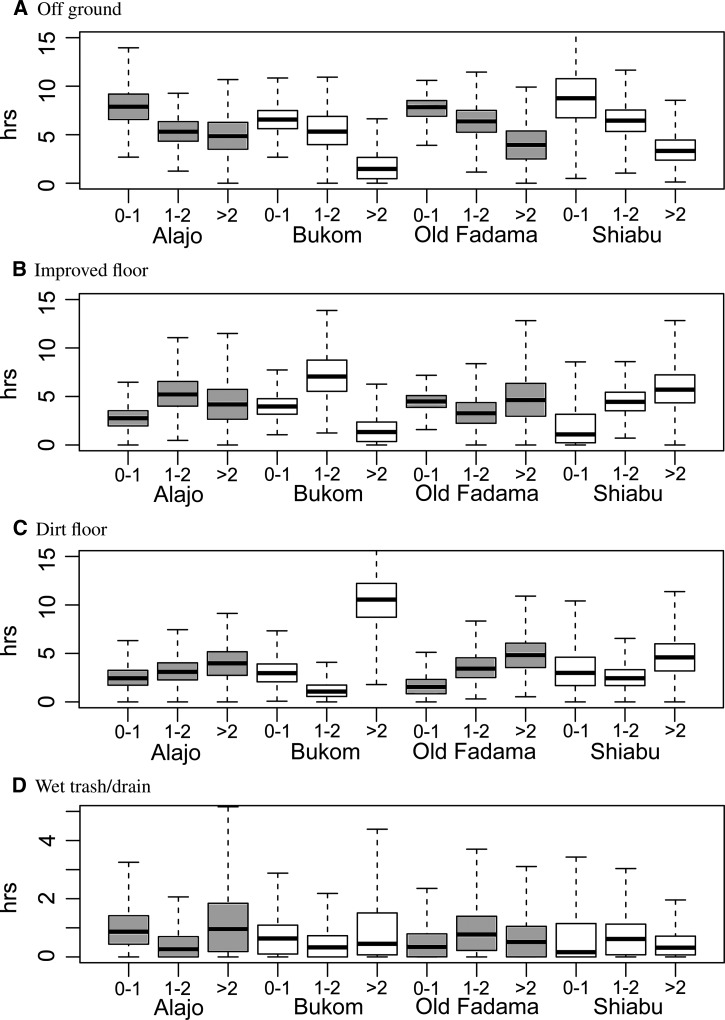
Estimated total times spent in four primary household compartments, by child age and neighborhood.

). In contrast, the study children were rarely observed to have contact with drains and stagnant water/trash areas. The graphs for the four neighborhoods are similar, but not identical.

Simulated sequences of behavior were generated covering a complete waking period to represent time use of a child in a study neighborhood: what the child was doing and where this occurred. Some activities (playing, sleeping) are best characterized by their duration, accumulated over all occurrences during a daily waking period, whereas other more discrete activities (eating, handwashing, bathing, defecation) are better characterized by the frequency with which they occur.

**Figure 7.**
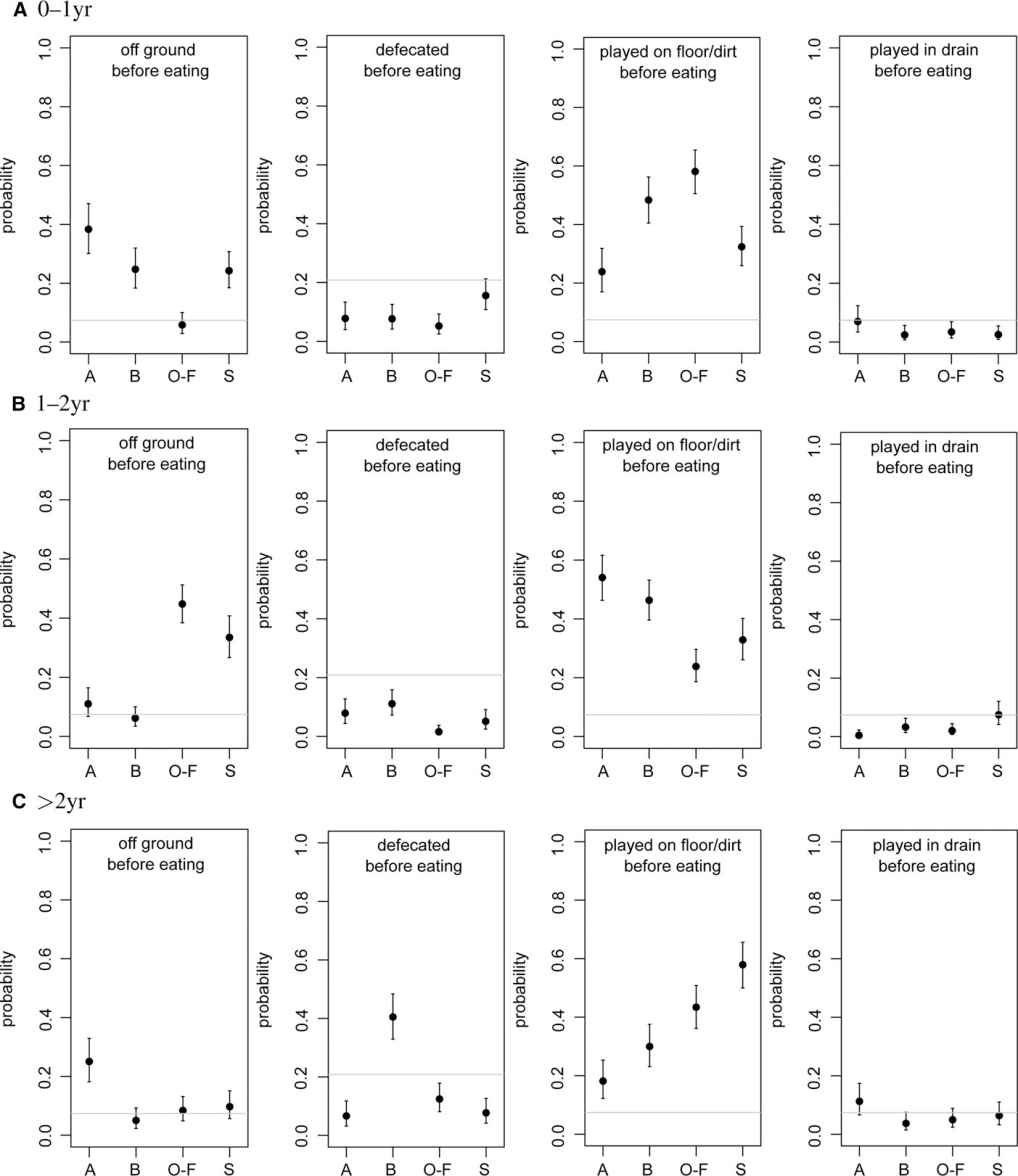
Probabilities of behavioral sequences, from simulated states of children less than 5 years of age in households in the four neighborhoods: (A)lajo, (B)ukom, (O)ld(-F)adama, and (S)hiabu. Graphs show means and 95% ranges from *N* = 1,000 simulations; the horizontal line is a reference level from an unweighted network (see “Results” section). From left to right: off ground before eating, defecate before eating, playing on dirt/improved floor before eating, and playing in drain or wet trash area before eating.

The graphs in all remaining Figures 2–7

are based on simulated populations of 1,000 children in households, stratified by age and neighborhood.

Simulated behavioral sequences may be used to calculate descriptive statistics. Figure 2 shows marginal durations for children in various household compartments, stratified by age and neighborhood. Figure 3

**Figure 2.**
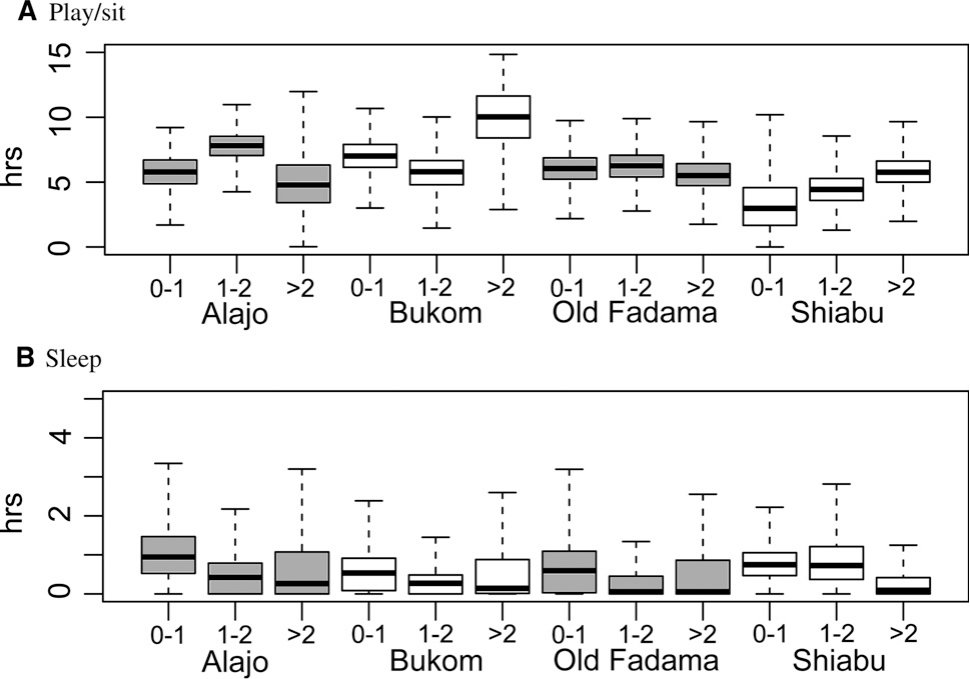
Estimated total duration of activities of children in households, by age and neighborhood. Note that there is sleeping at night that was not observed.

shows marginal durations of two main activities (playing/sitting and sleeping) and Figure 4

**Figure 4.**
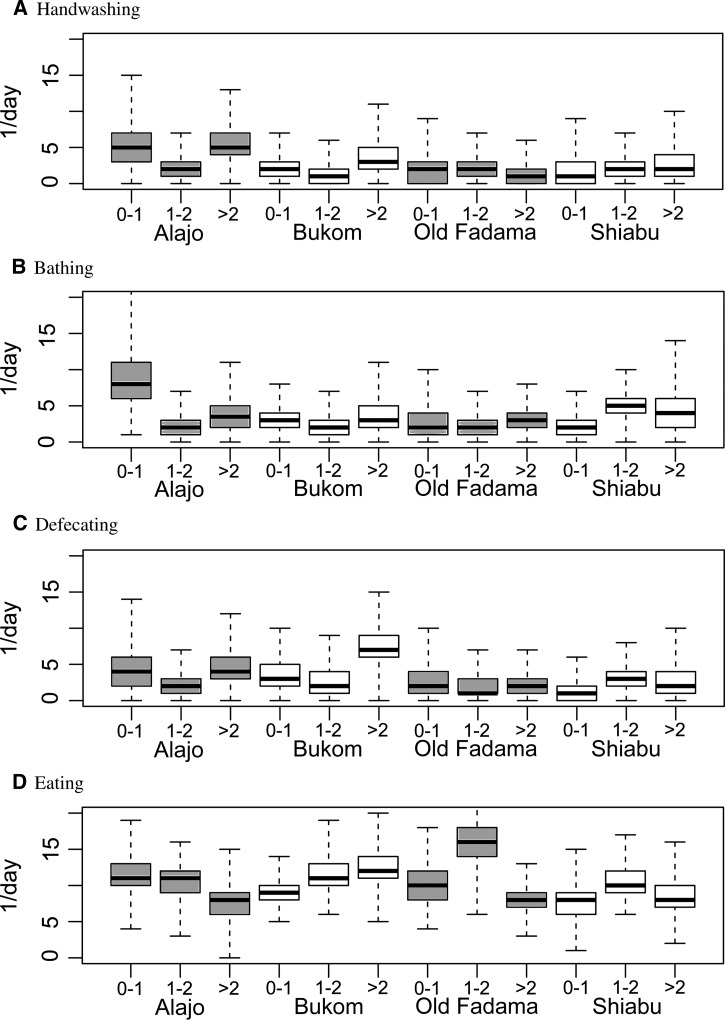
Estimated daily frequencies of activities of children in households, by age and neighborhood (the numbers of times these activities occurred in the simulated daily behavior sequences).

shows marginal frequencies of the four other activities (handwashing, bathing, defecating, and eating), ignoring the compartment where they occur. Children *>* 1 year spent less time off ground and more time on floors (in Bukom more time on dirt floors). Relatively little time was spent in drains or wet trash areas (note the different scale in Figure 2D). Younger children spent more time sleeping during daylight time. Playing was the predominant activity for all observed ages and comprised about half of the daylight time. Of the discrete activities, eating was most frequent. Times and frequencies of activities and the compartments where they occurred, can also be shown as matrix graphs (Supplemental Appendix, Supplemental Figures 8 and 9).

**Figure 5.**
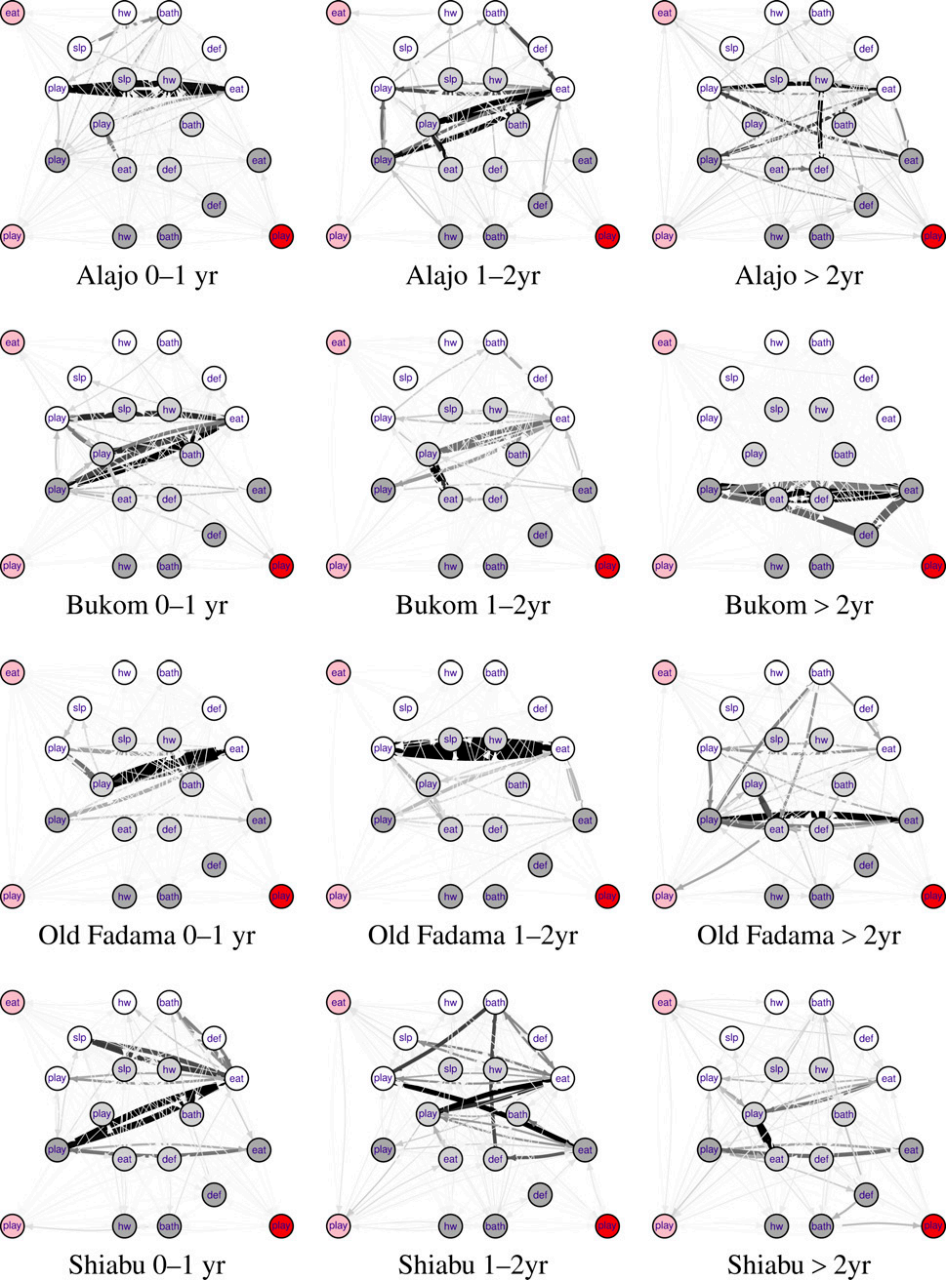
Simulated states of children (*N* = 1,000) less than 5 years of age in households in the four study neighborhoods, and simulated transitions among these states.

Just like the observed data, simulated sequences may be translated into network graphs. Figure 5

shows network graphs for simulated child behaviors. Clearly, the results show differences among neighborhoods, but Figure 5 also shows how the model differentiates between behaviors of children of different ages within each neighborhood.

In newborn infants (0–1 year), the transition from playing/sitting off ground to eating off ground and vice versa is most frequent in Alajo, while in Bukom going from playing/sitting on improved floor or dirt floor to eating off ground (and vice versa) also occurs commonly. In Old Fadama and Shiabu, playing/sitting off ground to eating off ground is not the most common transition. In older children, the transition from playing/sitting to eating is still frequently observed, but occurs in other compartments.

These simulated graphs show some rare states not present in the observed data. Any state that was observed in any neighborhood was deemed possible in any other neighborhood, and the estimated rate parameter for that state (or rather for that state and its ancestor) was kept, noting however that in neighborhoods where this state was absent, its rate was estimated to be low enough to make that absence likely.

**Figure 6.**
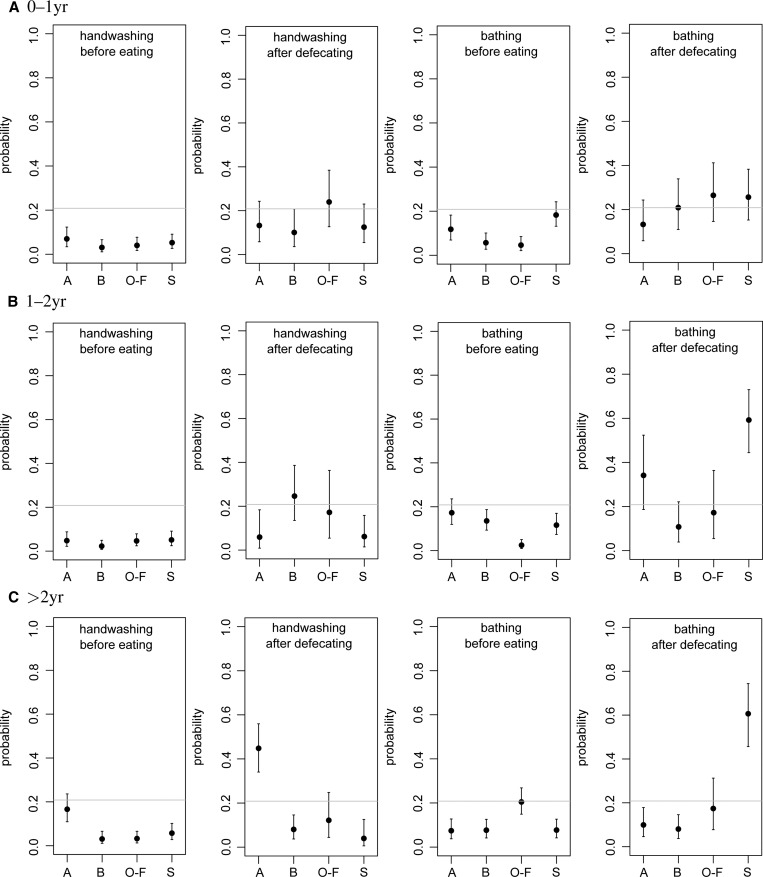
Probabilities of behavioral sequences, from simulated states of children less than 5 years of age in households in the four neighborhoods: (A)lajo, (B)ukom, (O)ld(-F)adama, and (S)hiabu. Graphs show means and 95% ranges from *N* = 1,000 simulations; the horizontal line is a reference level from an unweighted network (see “Results” section). From left to right: handwashing before eating, handwashing after defecation, bathing before eating, and bathing after defecation.

The network representations of the state diagrams are convenient for analyzing the probabilities that specific sequences of behavior occur, such as those that are of interest for risk of enteric infections. For instance, the probability of handwashing occurring before eating (incoming edges from handwashing to eating, in any compartment) may be calculated, as a fraction of the frequency of any other activity before eating (any other incoming edges into eating nodes, in any compartment). In Figures 6

and 7, the probabilities of selected sequences of behavior are shown. The horizontal line in these graphs is a reference level, calculated by using the same statistic on a completely uninformed network with all states present and all edges equally likely (all edge weights = 1).

Figure 6 shows that handwashing just before eating was very rare in all neighborhoods and all ages (except perhaps children *>* 2 years in Alajo). In fact, bathing before eating was slightly more likely than handwashing before eating. A similar conclusion may be drawn from looking at handwashing after defecation, counting defecation events succeeded by handwashing (outgoing edges from any defecation node toward any handwashing node), relative to those preceded by any other activity (outgoing edges from defecation nodes toward any other nodes), which appears only as likely as would happen if all states were equally likely. Indeed, bathing after defecation was slightly more likely than handwashing after defecation.

Figure 7 shows a few other relevant sequences: being off ground before eating (indicating smaller risk of contact with contaminated surfaces), defecating before eating, playing on floor or dirt floor before eating, and playing in drain or wet garbage area before eating.

## Discussion

In societies where sanitation is poor^17^–^19^ and where attitudes toward hygiene may not be informed by knowledge about disease transmission,^20^,^21^ any behavior that involves contact of a young child with fecal contamination implies a potential health risk.^22^ The ultimate goal of the study reported in this article is to contribute to studies of quantitative microbial risk assessment,^23^ by improving the information available on child behaviors related to contact with fecal matter. The presence and concentration of fecal contamination in each compartment may be characterized by environmental sampling and detection methods.

The probability of contact with, and ingestion of, fecal matter, associated with any activity, may be quantified using exposure factors,^24^ or specific data on transfer of microbes, for instance, from surface to hand and from hand to mouth.^25^–^32^ What is often missing in risk studies is a quantitative description of the occurrence of any contact behaviors based on empirical knowledge. The contribution of this study is to provide such information by the simulated sequences of states. Not only does this allow prediction of the time spent in any state, or the frequency with which any state occurs, this analysis also provides a faithful (data-based) prediction of the order in which behavioral states occur. Any activity that is associated with a high probability of picking up fecal contamination, on hands, is risky when it is followed by an activity that implies ingestion, for instance eating with dirty hands or mouthing of a dirty hand. The order in which contact events happen is the key to exposure risk.

Although the compartments that may be considered most likely to cause contact with fecal matter (wet garbage areas and drains) were visited less frequently than other compartments (Figure 2 and Supplemental Figures 5 and 9), they may be important for exposure to fecal pathogens because of the probable high concentrations of fecal matter, and because both are wet environments, potentially increasing the survival of microbes and the efficiency with which fecal pathogens are transferred to hands by physical contact.

The youngest children (0–1 year) spend more time off ground, presumably giving them less opportunity for picking up fecal matter from contaminated floors or other surfaces. However, our results also show that it is likely that a child of any age is in a contaminated compartment directly before eating activities, without handwashing.

Studies of diarrheal disease often show a correlation with socioeconomic status (SES).^33^ Comparing a “low SES” neighborhood (Old Fadama) with a “middle class” neighborhood (Alajo)^13^ does not reveal strong differences in child behavior, but the higher chance of infants less than 1 year of age being off ground in Alajo compared with the higher chance of infants of the same age playing on the floor or in the dirt in Old Fadama may suggest different behavioral patterns (Figure 2). There is also some evidence that children less than 1 year old in Alajo had their hands washed more frequently and were bathed more often than in the other neighborhoods.

The study reported here explores a method for quantifying behavior that has not previously been used in the context of exposure assessment. Although we have attempted to extrapolate where possible, the observations cannot provide a complete description of child behavior. All observations happened during daylight hours. The simulated behaviors therefore can be considered valid only for that part of the day: what children did during nighttime, and where they were, was not recorded. It is likely that most of that period was spent sleeping off ground, but young infants must have been fed during nighttime. Another limitation of the data collection for this study was that the definition of eating used in the structured observations, did not include breastfeeding, nor was drinking explicitly recorded. Given the required intake of food and drink in young infants, one may surmise that frequent drinking must have occurred, but unfortunately we have no specific data documenting this. We also do not have specific data recording breastfeeding because this activity was likely to have occurred indoors out of view of the observer, and we were not able to accurately record frequency and duration.

To limit the spectrum of observed behaviors to a manageable number, this study focused on child activities that cause oral exposure to fecal contamination from the environment and by design we did not record social interactions and physical contact between the study child and the caregiver, nor with other young children. For example, the enumerators did observe instances of groups of young children playing in open drains and playing with the study child, offering food that had been on the ground, and having close physical contact. Some activities of caregivers are relevant for children's exposure^34^ (like preparation of food or cleaning the room) and were recorded, but they cannot be directly linked to the states of the observed children. For that reason, we have not included caregiver behavior into the model. Although in some households more than one child was observed, interactions among children were also not recorded, so that we also cannot relate any change in the state of a child to the actions of other children in the same compartment. Future studies should expand observations to include such interactions among children and between children and their caregivers, so that these categories of behavior may also be quantified.

This study is based on prolonged observations of single children, aimed at collecting a complete record of their behavior. Observer effects influencing the behavior of the observed children may have been present,^35^,^36^ however, it is unknown to what extent such effects may occur in children aged 0–5 years. The observer usually spent several hours in a household or nursery, not interfering and quietly taking notes, so that any observer effects may be small and consistent among observation sessions. One may still argue that use of video recording^11^,^28^,^37^ could have made the observations more reliable, and in particular, could have offered opportunities for correcting oversights in retrospect. Other structured observation approaches,^31^ focusing on the range of hand–object–mouth pathways may also be useful for future extensions to better document how often specific contacts occur.

Competing risk models have been used for studies of human behavior,^38^ in activity scheduling related to travel, shopping, and other decision-making behavior. Our application of these models in combination with graphs to visualize and study sequences of behavior is novel to our knowledge. It is attractive to interpret the competing hazards model of changes in state as a description of the motivational state of a subject: with the start of any new activity and/or change in compartment, the motivation to move on to any successive state starts to increase, until the change happens and the process starts anew. From the time the child starts playing, its motivation to eat, sleep, wash hands, bathe, or defecate increases until one of the alternatives wins. Our data show that the winners are usually sleeping or eating.

Although we have determined the transition rates dependent on the current and the previous state, it may be considered a limitation that earlier states cannot be taken into account. Passage to a new state may be contingent on several previous states of an individual. The simple model we have used assumes that the path (through state space) is completely defined by the hazard rates associated with each activity and location (compartment), as well as the state that immediately preceded the present one: any child can perform any activity in any location at any time, and the probability of changing its location only depends on where it is, and on the location visited before the present one, not anything earlier. Such prior states may be important,^34^ for instance, when a child plays in an open drain before eating (and not washing hands). Expressing the behavioral data as directed weighted networks where states are nodes and transitions between states are the edges is useful for efficient analysis of patterns in behavior. A possible extension, as yet to be explored, is the use of network statistics (centrality metrics) to identify a variety of other patterns in behavior.

In conclusion, the competing hazards model allows quantitative analysis of time specific behavioral data, efficiently using the observed durations of states to infer time used for behaviors, frequencies of behaviors, and the probability that critical sequences of activities occur. Combined with data on microbial contamination, this information on sequences of behavior allows us to study transfer of fecal pathogens from the environment to human ingestion and assess the contributions of various competing pathways to oral exposure. Such information on the magnitudes of exposure through different pathways is important for decision making and the design of interventions to reduce childhood diarrhea and other adverse health outcomes associated with enteric infections.

## Supplementary Material

Supplemental Datas.

## Figures and Tables

**Table 1:** Compartments within the household/nursery environments, where activities occur, and repertoire of observed activities

Environment	Compartment	Activity
Household/nursery	Unimproved ground (dirt)	Play/sit
	Improved ground (floor)	Sleep
	Off ground (caregiver, chair)	Wash hands
	Stagnant water/trash area	Bathe
	Open drain	Defecate
		Eat

**Table 2:** Example: structure of the behavioral data

Observation number	1	2	3	4	5	6	…	*N −* 1	*N*
Compartment	1	3	2	2	2	4	…	5	1
Activity	1	6	4	6	1	1	…	3	2
Time (minutes)	10	14	16	17	19	23	…	347	360

For any individual study child, a set of observations was recorded that consisted of three variables: the activity that was performed (numbered 1–6 as in Table 1), the compartment where this activity took place (numbered 1–5 as in Table 1), and the time (since start of the observations) when this activity started.

**Table 3:** Numbers of study subjects by neighborhood, numbers of observations (minimum to maximum) per subject, and duration of observation period (minimum to maximum), for structured observations of behaviors in households and in nurseries in Accra

Neighborhood	Households			Nurseries		
	Number of subjects	Number of observation	Time observed (minutes)	Number of subjects	Number of observation	Time observed (minutes)
Alajo	35	3–22	117–330	8	5–11	127–250
Bukom	23	8–31	230–320	5	2–6	107–222
Old Fadama	37	1–26	21–321	7	5–12	112–235
Shiabu	34	6–28	132–330	5	1–11	5–240
