# Supplementary material for: Quantifying Contact with the Environment: Behaviors of Young Children in Accra, Ghana
Source: Am J Trop Med Hyg. 2016 Apr 6;94(4):920–31. doi: 10.4269/ajtmh.15-0417 (PMC4824240; doi:10.4269/ajtmh.15-0417)
Supplement: Supplementary file 1 [file SD12.pdf]

SUPPLEMENTAL APPENDIX

ESTIMATING RATES OF BEHAVIOR

The observer starts observing at time $t = 0$. If an activity happens with a rate $h(t)$, then the probability that activity occurs within an interval $(0, T)$ is 1 minus the probability that the activity has not occurred by time $t = T$:

$$\mathrm{F}(T) = 1 - \mathrm{e}^{-\int_{u=0}^{T} h(u)\mathrm{d}u} = 1 - \mathrm{e}^{-hT} \tag{1}$$

the latter part in case of a constant hazard rate h. Within any observation period, there are several different states that can be observed, presumably each with their own rate. Each time a new event takes place, the time is recorded, so that the result is a record of consecutive states and their starting times. During the k-th interval of the observation period, a child is in state s_k—one out of a total of N mutually exclusive possible states. If the time elapsed between start of state s_k and start of the next state is T_k, and the new state starting at the end of the interval $(0, T_k)$ is s_{k+1}, then the likelihood that this occurs is:

$$\mathrm{l}_{s_k \to s_{k+1}} = \left(h_{s_k \to s_{k+1}} \mathrm{e}^{-h_{s_k \to s_{k+1}} T_k}\right) \prod_{\substack{i=1 \\ i \neq s_k \\ i \neq s_{k+1}}}^{N} \mathrm{e}^{-h_{s_k \to i} T_k} \tag{2}$$

in case of a constant hazard h. This is the probability that the interval $(0, T_k)$ is ended by a transition to state s_{k+1} (and not to any other state), ignoring the present state. This is called a *competing hazards* model.[1,2] The hazard rates of leaving any state may depend on both the new state s_{k+1}, and the present state s_k.

Although the assumption that the hazard rates are constant is attractive because it is simple, it is unrealistic because of the exponential distribution of times between events: the shorter an interval, the more likely it is. This can be overcome by using a different hazard function. When (single event) intervals are distributed as

$$\mathrm{f}(T|r, \lambda) = \left(\frac{r}{\lambda}\right)\left(\frac{T}{\lambda}\right)^{r-1} \mathrm{e}^{-(T/\lambda)^r} \tag{3}$$

a Weibull distribution, then the hazard rate is

$$h(T|r, \lambda) = \frac{r}{\lambda}\left(\frac{T}{\lambda}\right)^{r-1} \tag{4}$$

and the survivor function (the probability that an event occurs within $(0, T)$ is

$$\mathrm{F}(T|r, \lambda) = 1 - \mathrm{e}^{-(T/\lambda)^r} \tag{5}$$

and the above likelihood function for the competing hazards of different alternative states becomes

$$\mathrm{l}_{s_k \to s_{k+1}} = \left(\frac{r}{\lambda_{s_k \to s_{k+1}}}\right)\left(\frac{T}{\lambda_{s_k \to s_{k+1}}}\right)^{r-1} \mathrm{e}^{-\left(T_k/\lambda_{s_k \to s_{k+1}}\right)^r} \prod_{\substack{i=1 \\ i \neq s_k \\ i \neq s_{k+1}}}^{N} \mathrm{e}^{-\left(T_k/\lambda_{s_k \to i}\right)^r} \tag{6}$$

assuming here that the shape parameter r is global: any activities in any compartments have the same shape parameter (but different scale parameter). For $r = 2$ or higher, short periods between events (transitions to different states) are unlikely, so that the intervals predicted by the Weibull model are more realistic than with the constant hazards model (Supplemental Figure 1).

Many observed sequences were censored: for the first observation in a sequence, the state preceding an observed state is unknown; the state after the last observation in a sequence is also unknown. During observation periods the child may be (carried) out of sight, which was recorded as missing. The likelihood function can be easily adjusted for such censored data (see below).

DEALING WITH CENSORED OBSERVATIONS

For a record of K observations of a single child, the log likelihood can be calculated:

1. $k = 1$ (first observed interval) old state unknown, new state (s_1) known; all outcomes left censored:

$$L_{\to s_1} = T_1^r \left(\left(\frac{1}{\lambda_{s_1 \to s_1}}\right)^r - \sum_{i=1}^{N}\left(\frac{1}{\lambda_{i \to s_1}}\right)^r\right) \tag{7}$$

2. $1 < k < K$: old state (s_k) and new state (s_{k+1}) known:

$$L_{s_k \to s_{k+1}} = \log(r) + (r-1)\log(T_k) + \log\left(\left(\frac{1}{\lambda_{s_k \to s_{k+1}}}\right)^r\right) + T_k^r\left(\left(\frac{1}{\lambda_{s_k \to s_k}}\right)^r - \sum_{i=1}^{N}\left(\frac{1}{\lambda_{s_k \to i}}\right)^r\right) \tag{8}$$

3. $k = K$ (last observed interval) old state (s_K) known, new state unknown; all outcomes right censored:

$$L_{s_K \to} = T_K^r\left(\left(\frac{1}{\lambda_{s_K \to s_K}}\right)^r - \sum_{i=1}^{N}\left(\frac{1}{\lambda_{s_K \to i}}\right)^r\right) \tag{9}$$

For this observation, the log likelihood then becomes

$$L = L_{\to s_1} + \left(\sum_{k=2}^{K-1} L_{s_k \to s_{k+1}}\right) + L_{s_K \to} \tag{10}$$

This calculation can then be repeated for other children, over the four study neighborhoods.

PARAMETER ESTIMATION

The rate parameters for leaving states may be estimated by utilizing the log likelihood function (Equation 10). As a simplifying assumption, the Weibull shape parameter was kept at a constant value $r = 4.482$ ($\log(r) = 1.5$) so as to avoid intervals shorter than a minute.

The remaining 20 states, with 20×20 corresponding rate parameters, were estimated using a Bayesian framework, implemented in JAGS/rjags, by explicitly calculating the log–likelihood function (Equation 10). The prior used for $\log(\lambda)$ was a normal distribution with mean 6.0 and precision 0.1, assigning a low rate to unobserved transitions. JAGS source

code for estimating rate parameters for the competing hazards model can be supplied by the first author.

SIMULATING SEQUENCES OF BEHAVIOR

To simulate behaviors, the competing hazards model may be used as follows:

Starting from a specific state (compartment, activity)

1) Choose a random iteration from the Monte Carlo sample of (posterior) estimates of hazard rate parameters, representing a single child.
2) From estimated rates of all (admissible) states, generate a random sample using the appropriate Weibull distribution. This defines a set of time intervals to the end of the present state for each admissible next state.
3) Choose the new state with the shortest time interval to determine the next state (new compartment–activity combination).
4) And repeat this sequence as often as necessary, for example, to fill a specific period of time (Supplemental Figure 2).

Repeat this sequence of simulated states as often as the number of children needed for the simulation.

REFERENCES

1. Prentice RL, Kalbfleisch JD, Peterson AV, Flournoy N, Farewell VT, Breslow NE, 1978. The analysis of failure times in the presence of competing risks. *Biometrics 34:* 541–554.
2. Martinussen T, Scheike TH, 2006. *Dynamic Regresssion Models for Survival Data. Series: Statistics for Biology and Health*. New York, NY: Springer.

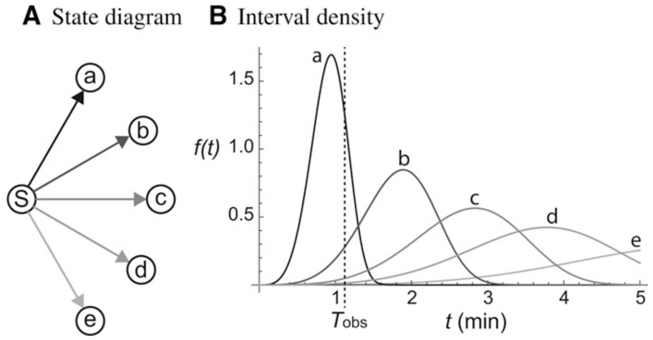

SUPPLEMENTAL FIGURE 1. Competing hazard model: (**A**) from state S, the child may move to any of the connected states a, . . . , e. Each of these transitions occurs with its own hazard rate, with corresponding interval density (**B**). In case the observed interval is T_{obs}, state a is most likely, state b is not much less likely, while the remaining states c, d, e are increasingly unlikely.

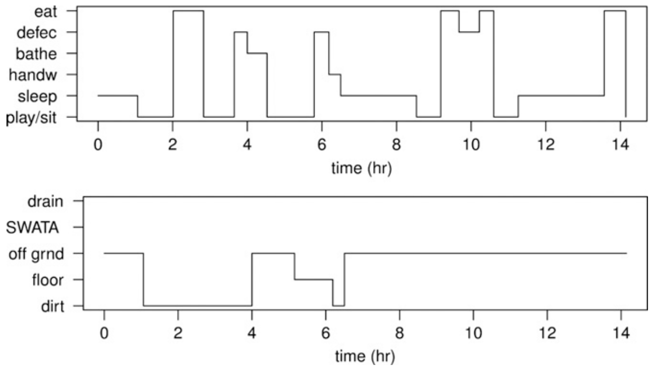

SUPPLEMENTAL FIGURE 2. A “day in the life of a child”: simulated random walk through state (activity–compartment) space. Example of a daily sequence for a child in Shiabu neighborhood, Accra.

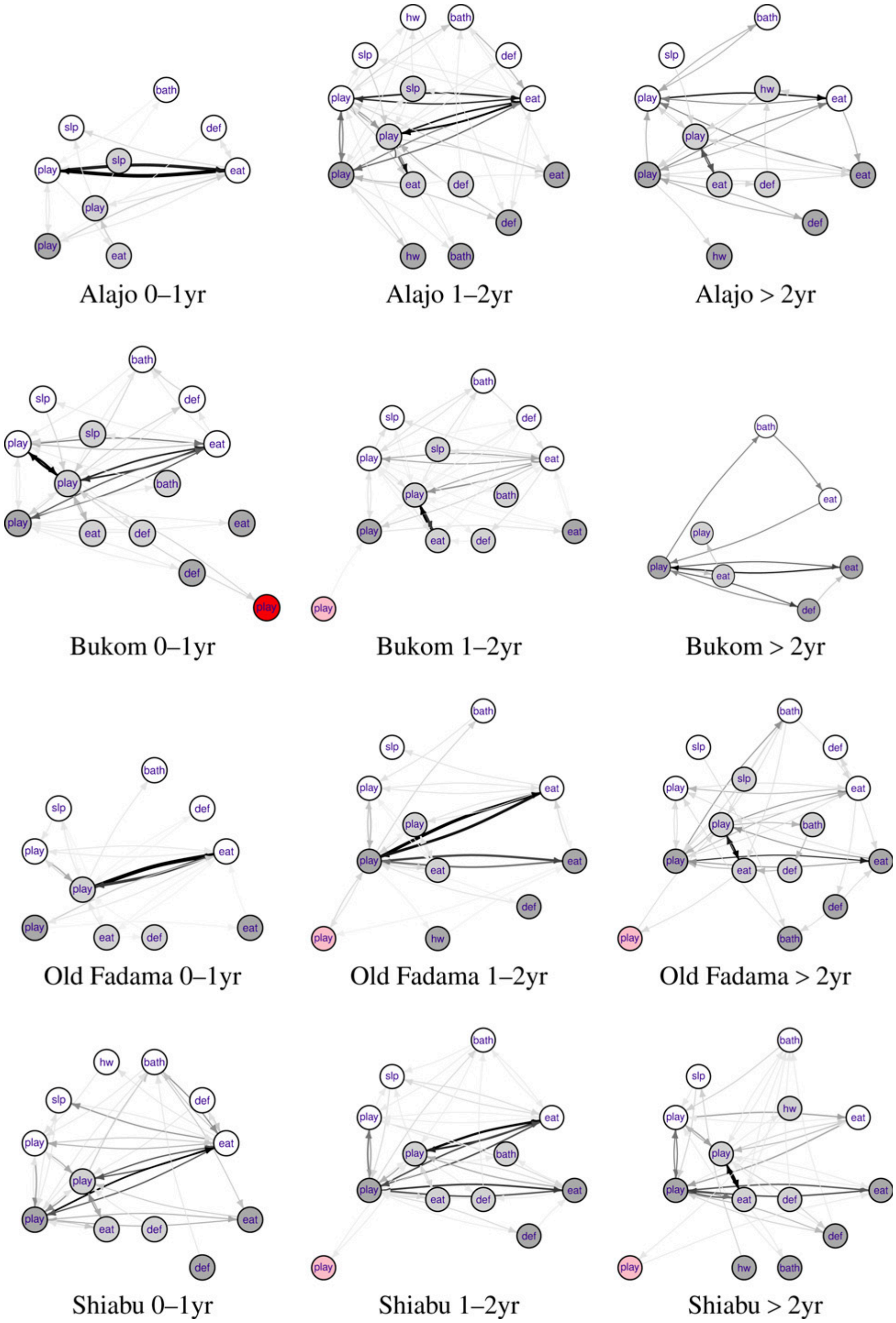

SUPPLEMENTAL FIGURE 3. Observed states of children less than 5 years of age in households in the four study neighborhoods.

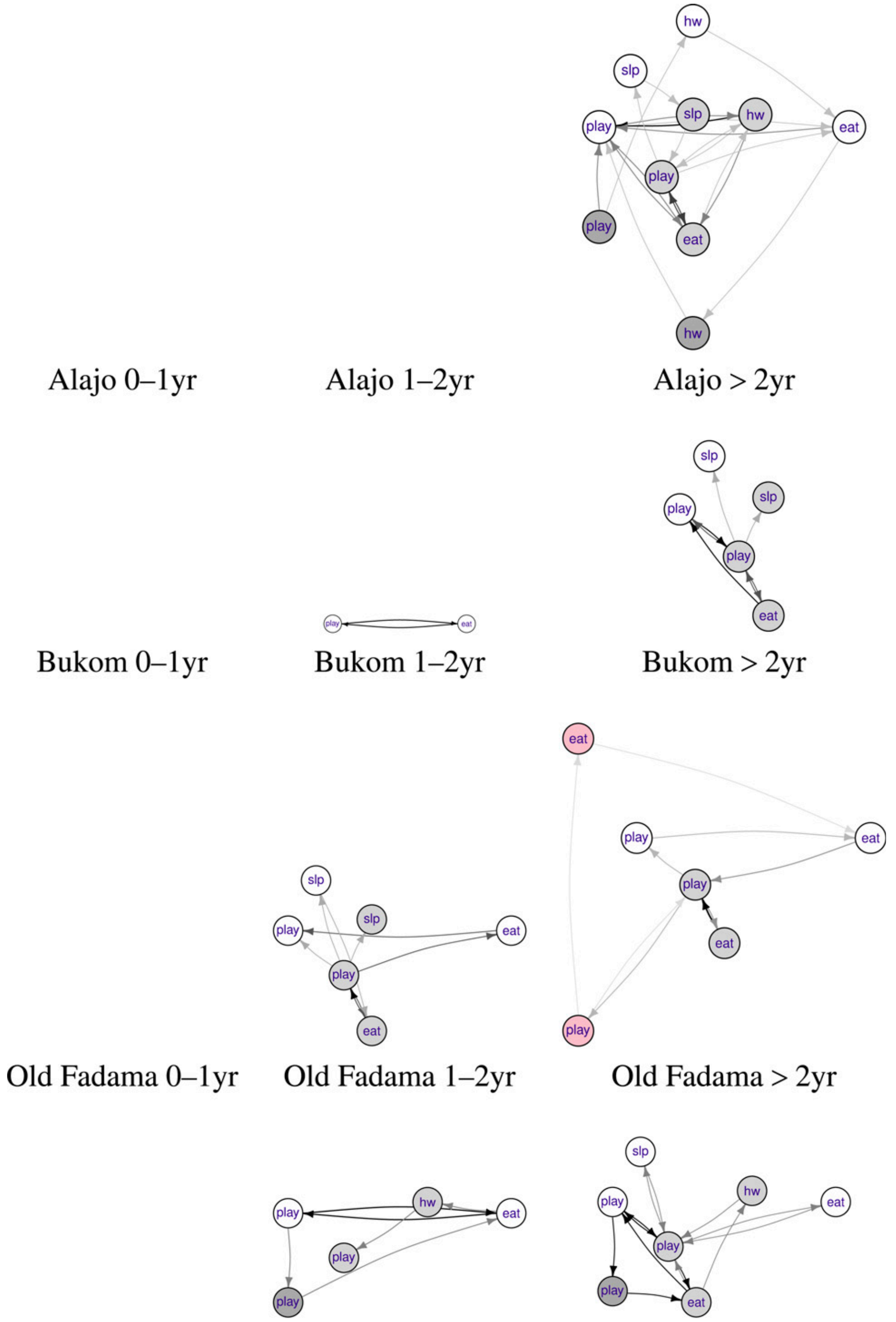

Supplemental Figure 4. Observed states of children less than 5 years of age in nurseries in the four study neighborhoods. Note that several categories are missing.

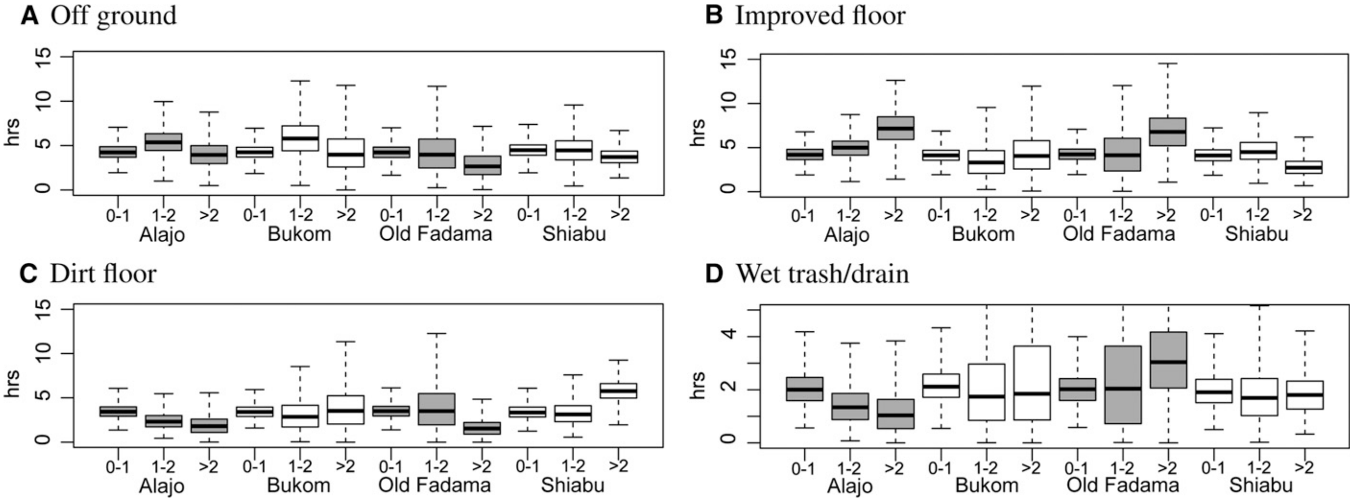

Supplemental Figure 5. Total times spent in four primary nursery compartments, by child age and neighborhood.

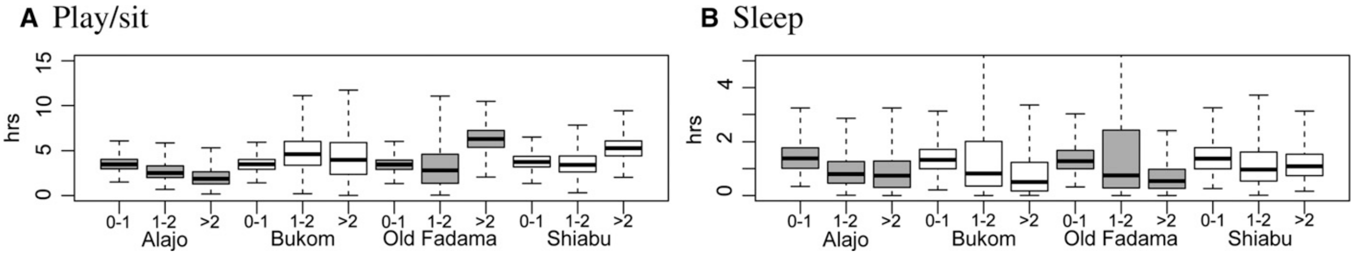

Supplemental Figure 6. Total duration of activities of children in nurseries, by age and neighborhood. Note that there is sleeping at night that was not observed.

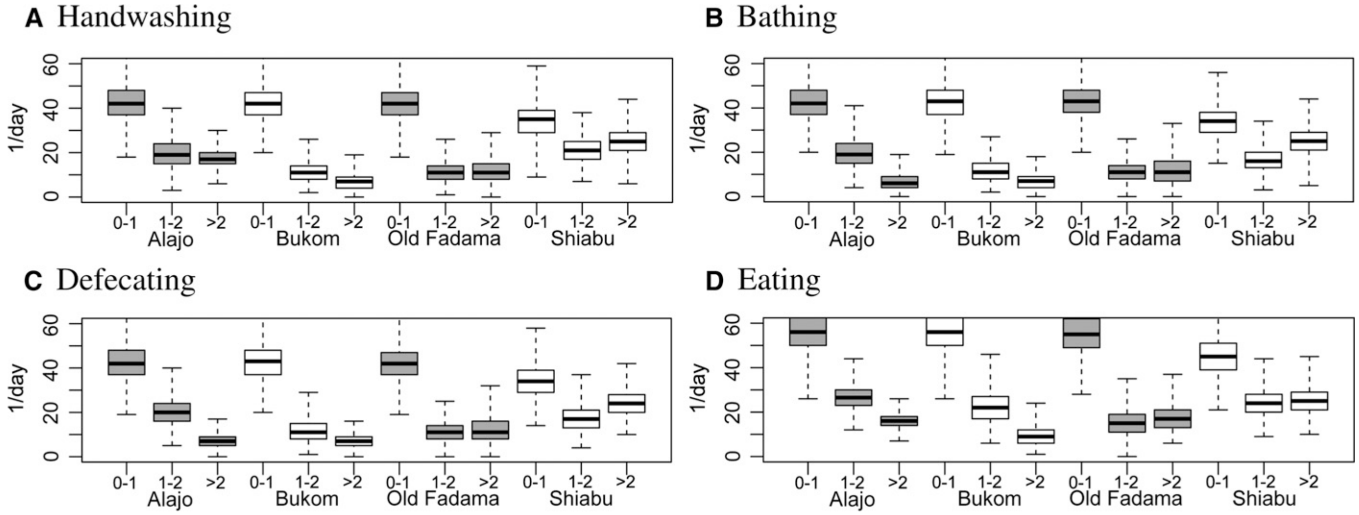

Supplemental Figure 7. Daily frequencies of activities of children in nurseries, by age and neighborhood (the numbers of times these activities occurred in the simulated daily behavior sequences).

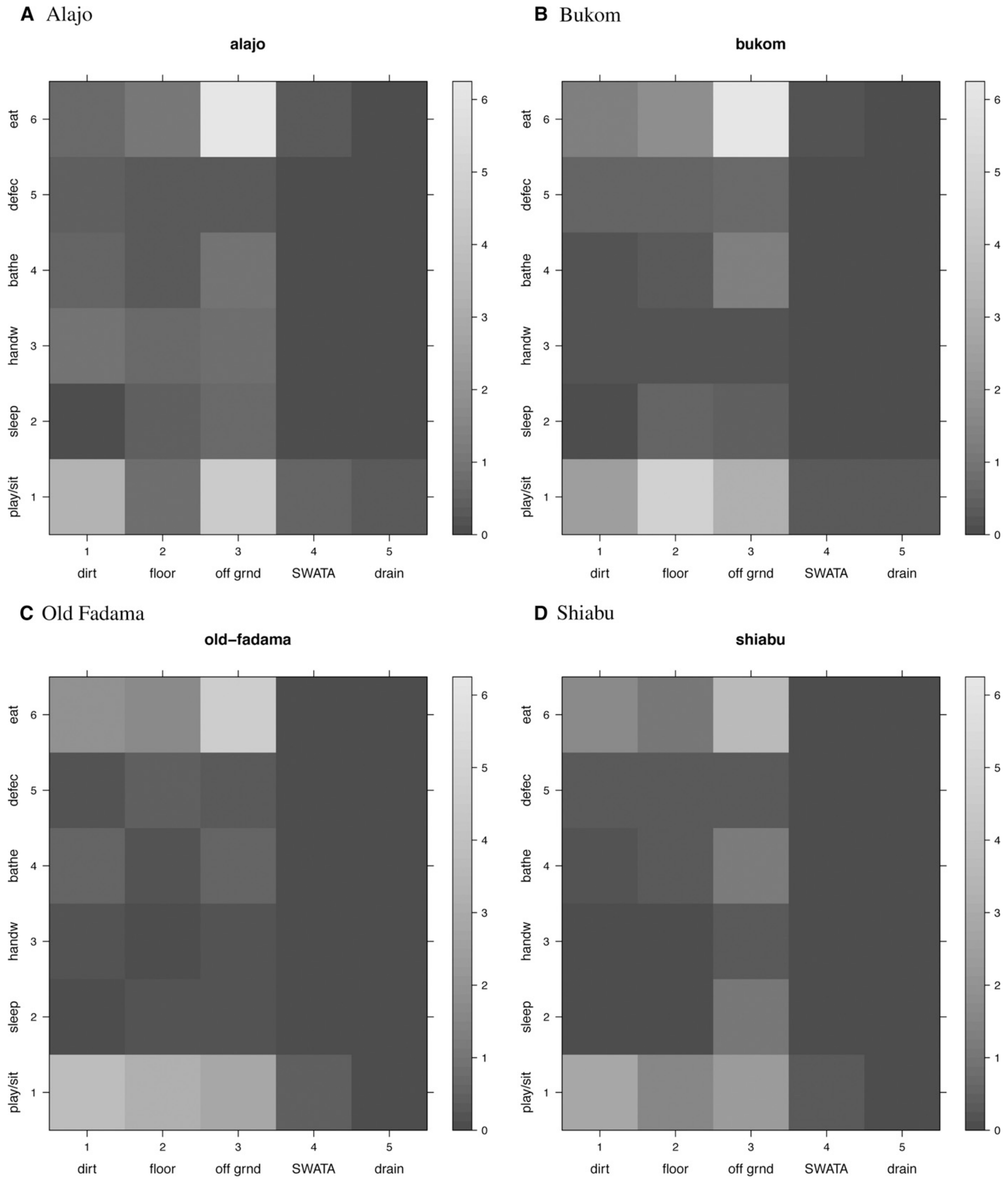

SUPPLEMENTAL FIGURE 8. Matrix plot of average frequencies of states (numbers of times per day) in households.

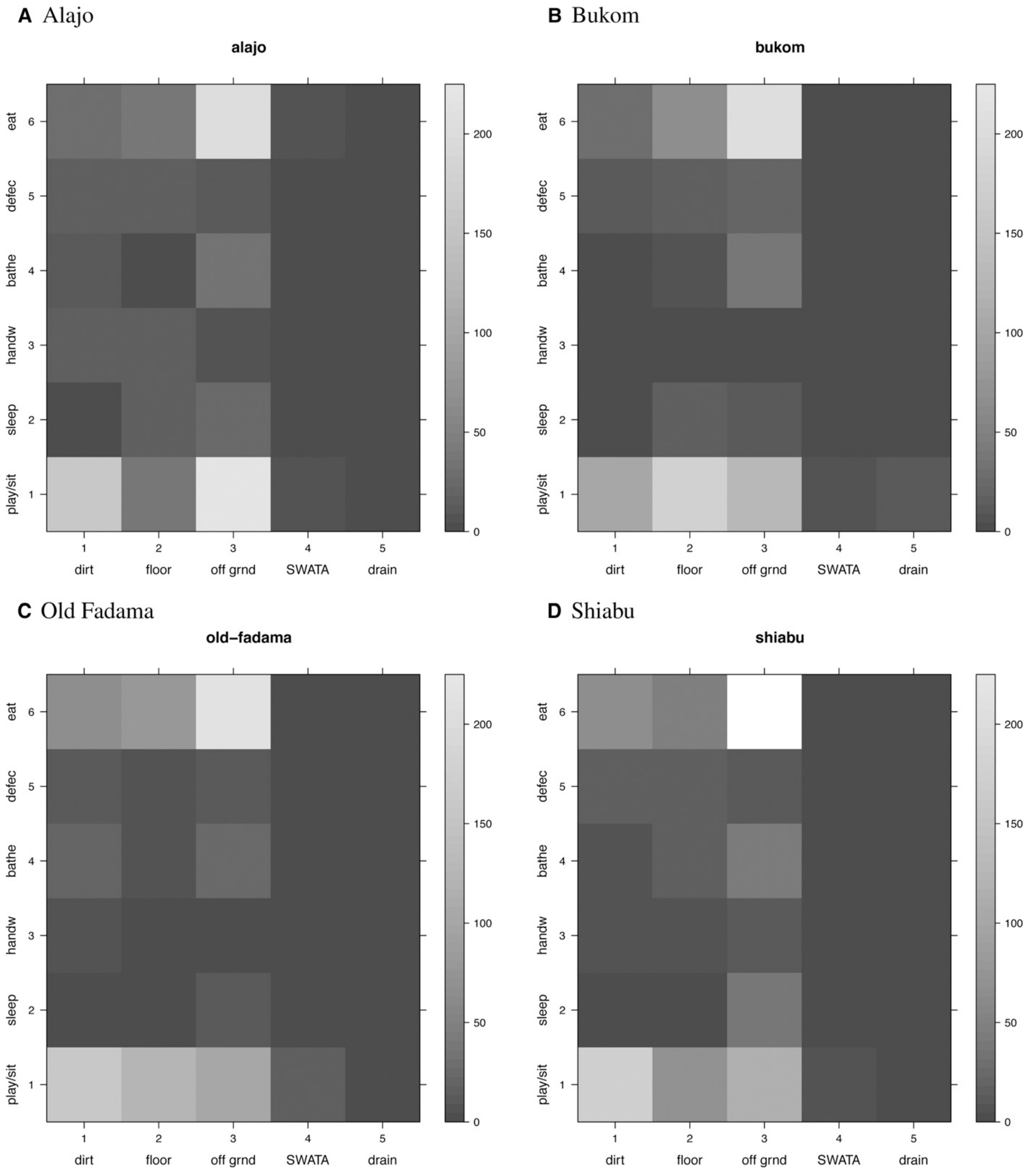

Supplemental Figure 9. Matrix plot of average times spent in states (numbers of hours per day) in households.

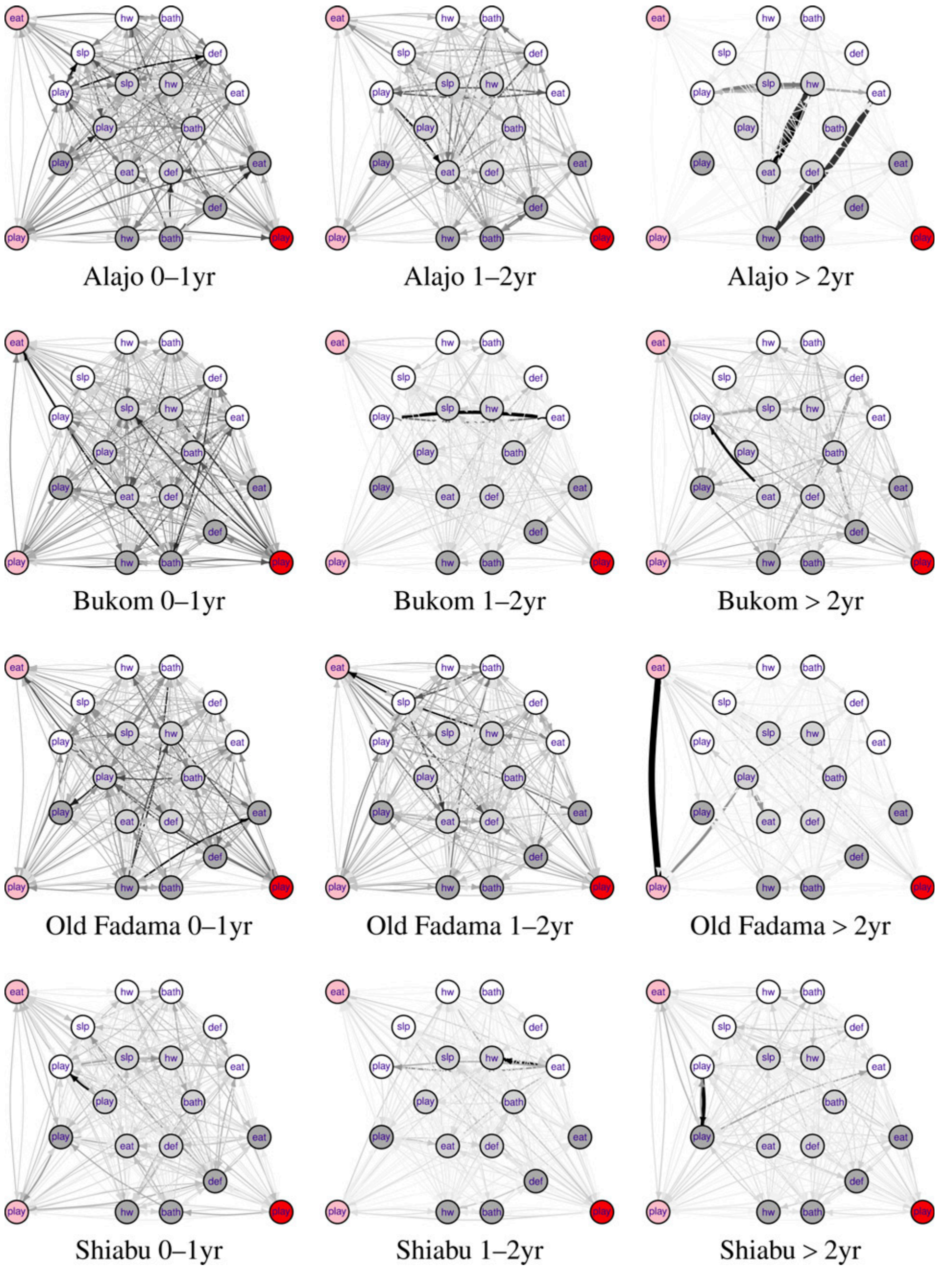

Supplemental Figure 10. Simulated states of children (N = 1,000) less than 5 years of age in nurseries in the four study neighborhoods, and simulated transitions among these states.

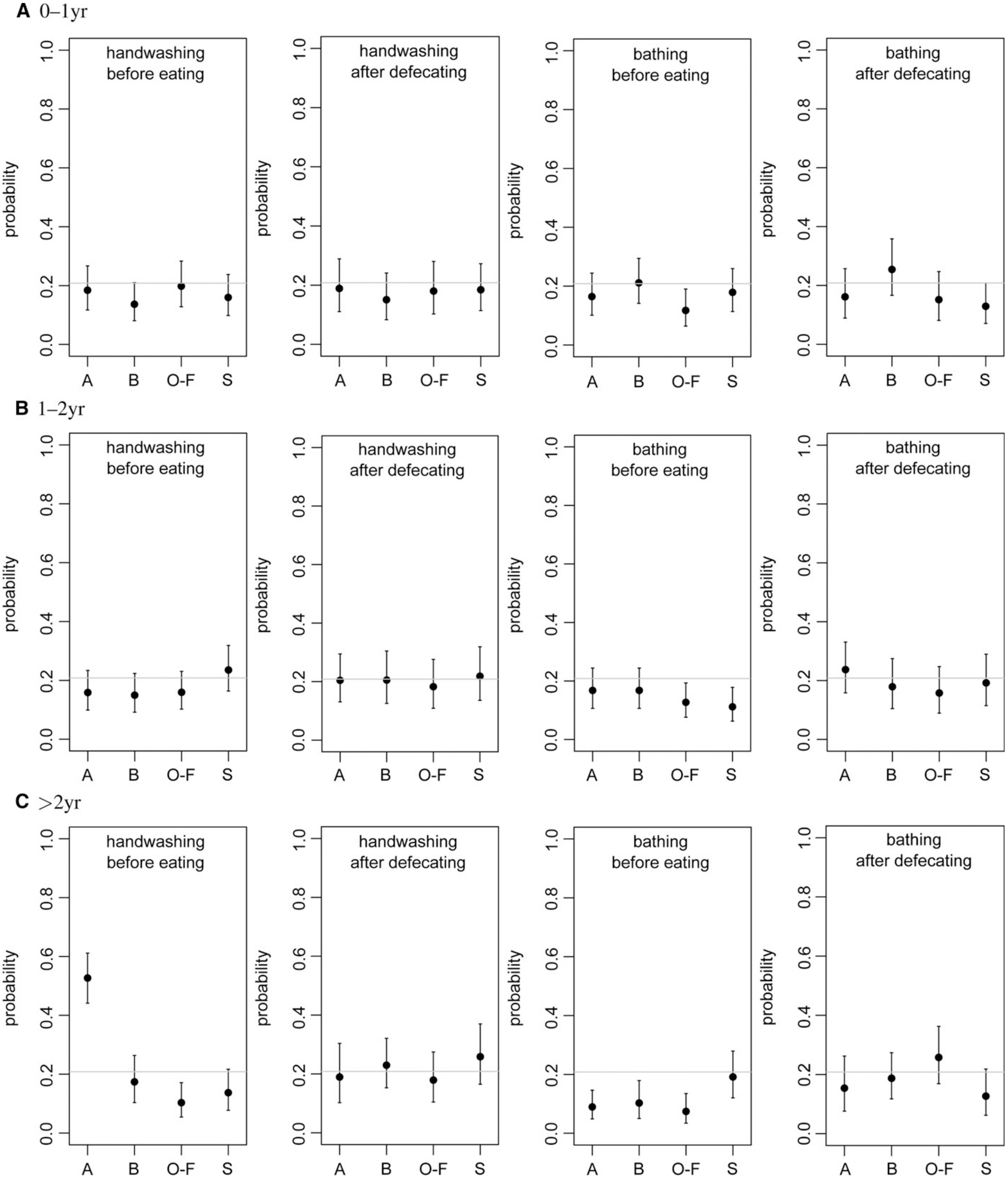

SUPPLEMENTAL FIGURE 11. Probabilities of behavioral sequences, from simulated states of children (N = 1,000) less than 5 years of age in nurseries in the four neighborhoods: (A) lajo, (B)ukom, (O)ld(-F)adama, and (S)hiabu. From left to right: handwashing before eating, handwashing after defecation, bathing before eating, and bathing after defecation.

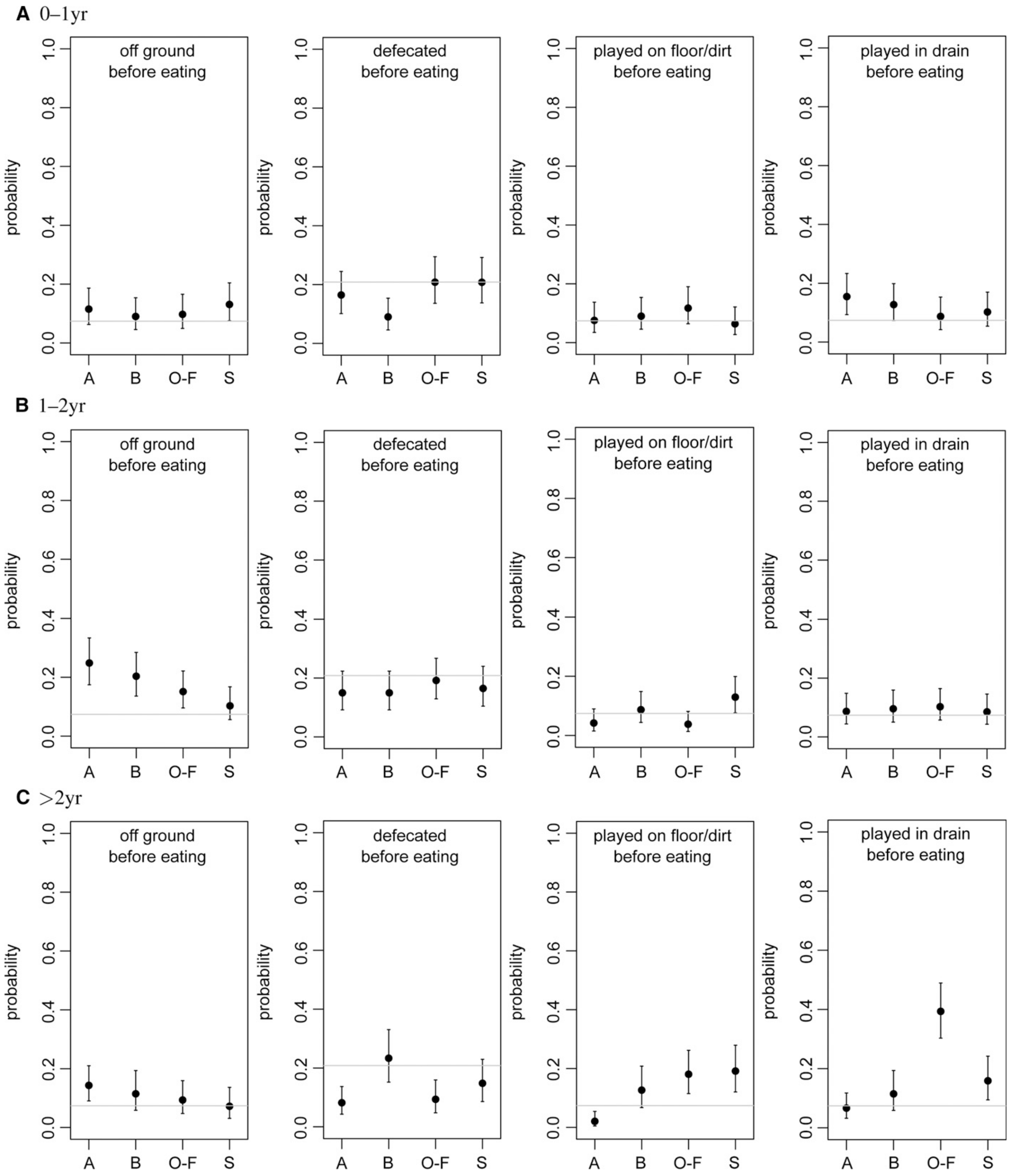

Supplemental Figure 12. Probabilities of behavioral sequences, from simulated states of children (N = 1,000) less than 5 years of age in nurseries in the four neighborhoods: (A) lajo, (B)ukom, (O)ld(-F)adama, and (S)hiabu. From left to right: off ground before eating, defecate before eating, playing on dirt/improved floor before eating, and playing in drain or wet trash area before eating.
